# Being trans in Korea: key milestones and stigmatization across life stages in a nationwide survey of 585 transgender and non-binary young adults

**DOI:** 10.4178/epih.e2025032

**Published:** 2025-06-27

**Authors:** Sungsub Choo, Ranyeong Kim, Hyemin Lee, Horim Yi, Rockli Kim, Seung-Sup Kim

**Affiliations:** 1 School of Public Health, San Diego State University, San Diego, CA, USA; 2 Policy Research Team, Department of Policy Research, Policy Headquarters, Korea Disabled People’s Development Institute, Seoul, Korea; 3 Department of Psychology, University of Maryland, College Park, MD, USA; 4 Solidarity for LGBT Human Rights of Korea, Seoul, Korea; 5 Interdisciplinary Program in Precision Public Health, Department of Public Health Sciences, Graduate School of Korea University, Seoul, Korea; 6 Division of Health Policy and Management, Korea University College of Health Science, Seoul, Korea; 7 Department of Environmental Health Sciences, Graduate School of Public Health, Seoul National University, Seoul, Korea

**Keywords:** Transgender persons, Sexual and gender minorities, Anti-transgender stigma, Transphobia, Developmental milestones

## Abstract

**OBJECTIVES:**

Understanding the experiences of transgender and non-binary (TGNB) individuals from a life-course perspective is essential. This article aims to identify ages at key milestones related to transgender identity and assess experiences of stigmatization among TGNB individuals across different life stages.

**METHODS:**

We analyzed data from a nationwide longitudinal survey of 585 TGNB adults in Korea collected in 2020 and 2021. Analysis of variance and chi-square test were used to compare mean ages at transgender identity developmental milestones and experiences of anti-transgender stigma across transgender identities.

**RESULTS:**

On average, TGNB Koreans realized their transgender identity at age 13, accepted it at age 20, and came out to others at age 21. Regarding experiences of stigmatization across different life stages, 67.4% reported hearing homophobic and transphobic remarks from teachers in secondary school. Among TGNB individuals assigned male at birth (AMAB) who served mandatory military service, 29.0% were classified as maladjusted soldiers, and 12.1% experienced sexual harassment or violence. When asked about the job application process, 57.0% reported discontinuing their job search due to their transgender identity. Financial burden represented the primary barrier to psychiatric evaluations, hormone treatment, and gender-affirming surgery.

**CONCLUSIONS:**

Our study identifies key milestone ages related to transgender identity and experiences of stigmatization across life stages among TGNB adults in Korea. With 97.6% of participants under 40 years old, these findings primarily reflect the experiences of TGNB young adults. Further research is necessary to better understand the experiences of middle-aged and elderly TGNB individuals in Korea.

## GRAPHICAL ABSTRACT

[Fig f2-epih-47-e2025032]

## Key Message

The average age of developmental milestones related to transgender identity include realizing their transgender identity at age 13, accepting it at age 20, and first coming out at age 21. TGNB individuals faced stigma and discrimination in key settings and situations such as secondary school, the military, and job applications from their adolescence and onwards.

## INTRODUCTION

“Transgender” is an umbrella term that encompasses gender identities that differ from the sex assigned at birth [1]. Some individuals identify as either a man or a woman (i.e., a trans man or trans woman) within the binary system of gender, while others do not. The latter are referred to as “non-binary” to acknowledge identities that challenge or defy the binary understanding of gender [2]. Transgender and non-binary (TGNB) individuals represent an important group who are expanding society’s understanding of gender and its expression. While their diverse experiences cannot be simplified into a monolithic characterization, TGNB individuals often face similar experiences of social stigmatization for transgressing societal gender norms and expectations [1-3].

Transgender identities and rights have become increasingly visible and heatedly debated in Korea, particularly following several high-profile cases in 2020. In January, Sergeant Hee-soo Byun publicly came out as transgender in protest of the Korean Army Headquarters’ decision to discharge her against her will following gender-affirming surgery [4]. As the first publicly known case of a transgender soldier in Korea, she subsequently died by suicide. In February 2020, a trans woman admitted to a women’s college withdrew her acceptance due to threats made online and offline in response to her acceptance [5]. These cases highlight the stigmatization Korean TGNB individuals may face in military and educational settings.

These examples of anti-transgender stigma reflect the broader context of the cisnormative culture prevalent in Korean society. Cisnormativity refers to the belief that gender is exclusively categorized as man or woman, determined at birth, and immutable throughout life. This expectation normalizes the experiences of individuals whose gender identity aligns with their sex assigned at birth (cisgender people), at the expense of erasing and stigmatizing those deviating from this norm—TGNB individuals [3]. As a cultural value, cisnormativity shapes policies and social environments, including those that are foundational to adolescent and early adult development. Between ages 14 and 19, Koreans must attend compulsory middle school, and free high school education is also provided. Following secondary education, individuals assigned male at birth (AMAB) are required to enlist in military service, typically in their early 20s. Upon their discharge from military service and graduation from college, Koreans enter the job market. Throughout these key phases of development and socialization, some of which are legally enforced, TGNB Koreans face challenges related to their gender identities.

These phases intersect with developmental milestones for transgender identity—distinct periods during which individuals form and realize their identities [6,7]. In a United States-based study of 26,957 TGNB adults, Tatum et al. [6] reported the mean ages for 3 transgender identity milestones: recognizing that one’s gender differs from one’s assigned sex (ages 8.35-12.82 years); identifying as transgender (ages 14.25-17.79 years); and first coming out as transgender (ages 20.84-29.73 years). These findings highlight adolescence and young adulthood as critical periods for transgender identity development [6,7]. TGNB individuals may be excluded from key developmental milestones and face discrimination and victimization when diverging from societal norms.

In societies that uphold and institutionalize cisnormative values, TGNB individuals experience health disparities compared to their cisgender counterparts [8,9]. Discrimination and victimization stemming from anti-transgender stigma accumulate in various social situations and settings throughout transgender individuals’ lives, leading to increased health burdens. Despite widespread experiences of discrimination across TGNB individuals’ lifespans, Korea lacks a national-level anti-discrimination law that includes gender identity and expression as protected categories [10]. Moreover, although studies have examined TGNB experiences in schools [11], healthcare settings [12-14], and employment [15,16], no reports have yet explored these challenges from a life-course perspective.

This study aims to identify the mean milestone ages related to transgender identity development among Korean TGNB individuals and to examine experiences of anti-transgender stigma across various life stages, particularly in schools, military service, job applications, and healthcare settings. Based on these findings, the present study provides implications for policy interventions designed to mitigate the anti-transgender stigma prevalent throughout Korean society.

## MATERIALS AND METHODS

### Data and participants

This study utilized data from the “Rainbow Connection Project III—Korean Transgender Adults’ Health Panel Survey,” collected from October 7-31, 2020 (Wave 1), and again during the same period in 2021 (Wave 2). This dataset represents a nationwide longitudinal survey with a non-probability sample of TGNB adults. Eligibility criteria included: (1) being over 18 years old; (2) holding Korean citizenship and residing in Korea; and (3) identifying as TGNB. Participants could join the survey through various online and offline channels, including: (1) Facebook pages and online transgender communities; (2) clinics specializing in gender-affirming healthcare services; (3) lesbian, gay, bisexual, transgender, and queer (LGBTQ) rights organizations; (4) friends and acquaintances; and (5) other channels.

To verify participants’ TGNB identities, the Wave 1 survey initially asked whether they self-identified as transgender, defined as including non-binary identities. Participants then answered 2 questions about their current gender identity (man, woman, or non-binary) and sex assigned at birth (male or female), following the “2-step” method of assessing transgender identity [17]. Participants whose gender identity differed from their sex assigned at birth were considered to meet the eligibility criteria.

After excluding 6 participants who did not respond to demographic questions, we included 585 responses from Wave 1. Of the Wave 1 participants, 483 agreed to participate in follow-up, and 390 initiated the Wave 2 survey after providing online consent. After removing duplicate entries (n=3), incomplete surveys (n=64), and surveys lacking answers to items on developmental milestones (n=5), 318 responses from Wave 2 were included in the analysis. A flowchart of participant inclusion is provided in the Supplementary Material 1.

### Measures

#### Transgender identities

Participants’ gender identities and demographic characteristics were collected in Wave 1. Using the 2-step process [17], participants could be categorized based on their sex assigned at birth as well as their current gender identity. Combining responses to sex assigned at birth and gender identity (man, woman, or non-binary), participants could be grouped into 4 categories: trans men, trans women, non-binary assigned female at birth (AFAB), and non-binary AMAB. Given the cisnormative culture of Korean society, which labels and socializes individuals based on the sex assigned at birth, categories identifying TGNB individuals’ gender identities on a spectrum relative to their sex assigned at birth were also utilized. These categories include transmasculine (i.e., AFAB individuals identifying on the masculine spectrum) and transfeminine (i.e., AMAB individuals identifying on the feminine spectrum). These 2 categorization systems, differing in their consideration of non-binary identities, were associated with distinct experiences among TGNB individuals.

#### Socio-demographic characteristics

In addition to transgender identity, the following socio-demographic characteristics were assessed in Wave 1: sexual orientation, age, highest level of education attained, employment status, monthly income, and region. Participants were also asked about their method of accessing the survey link (i.e., data collection channel) in Wave 1.

#### Transgender identity developmental milestones

We adapted a measure of developmental milestones specific to TGNB individuals from Tatum et al. [6] and incorporated it for Wave 2. Participants reported the ages at which they: (1) first thought they were transgender (regardless of their knowledge of the term “transgender”); (2) accepted their transgender identity (again, regardless of knowledge of the specific term); and (3) first came out to others as transgender. Ages for these developmental milestones were analyzed continuously.

#### Experiences of anti-transgender stigma across different settings

Experiences of anti-transgender stigma throughout the life course of TGNB individuals were measured at Wave 1. The survey investigated stigma stemming from policies, environments, and interactions with others in secondary schools, the military, and job application processes. For each setting, specific items were asked of participants who reported having attended secondary schools (n=579), AMAB participants who were serving or had completed mandatory military service (n=107), and participants who had applied for jobs in the past 5 years (n=467).

Regarding secondary school experiences, participants were asked about difficulties with sex-segregated physical environments (e.g., single-sex schools, single-sex classes, and single-sex dormitory assignments) and policies (e.g. wearing gendered uniforms), as well as anti-transgender victimization by teachers.

For mandatory military service experiences, participants responded to 2 items addressing difficulties (e.g., using communal showers and fear of being outed) and discriminatory practices (e.g., classification as maladjusted soldiers and referral to “Green Camp,” a specialized training program designed for soldiers facing adjustment challenges) related to their transgender identity.

In terms of job search experiences, participants initially answered a yes/no question about whether they had given up applying for a job due to their transgender identity. They were subsequently asked about other difficulties experienced by TGNB individuals during the job application process, including the cancellation or refusal of job offers. Participants were asked to select all applicable responses for items within each setting of experiences of anti-transgender stigma, resulting in mutually inclusive answers.

#### Use of gender-affirming medical interventions and reasons for non-use

Additionally, the survey explored participants’ experiences with seeking and receiving gender-affirming medical intervention (GAMI) and reasons for not receiving such intervention at Wave 1, informed by prior research indicating that barriers to GAMI represent a key form of structural stigma for transgender Koreans [12]. GAMI was considered to include the psychiatric diagnoses required to access further GAMI, hormone treatment, and gender-affirming surgery. Participants who had not used GAMI were asked to select all relevant reasons for non-use, and the analysis included the 3 most frequently endorsed reasons of non-use for each type of intervention.

#### Statistical analysis

Frequencies and proportions were calculated for participant characteristics and experiences of anti-transgender stigma across transgender identity categories. These categories were applied based on the significance of social experiences influenced by sex assigned at birth and the role of non-binary identity. Socio-demographic characteristics, experiences of stigma in secondary schools, and experiences of stigma during the job search process were reported using transfeminine/transmasculine categorizations, given that these characteristics were considered to be primarily affected by social experiences related to one’s sex assigned at birth. Analyses of transgender identity development, experiences of stigma in the military, and use of GAMI employed gender categorizations that distinguished non-binary participants, because being non-binary as opposed to binary transgender (i.e., trans women and trans men) was likely influential in gender identity development, gender socialization, and preferences regarding GAMI use.

Mean ages at milestones for transgender identity development were compared across all 4 identity categories using analysis of variance (ANOVA). Bonferroni post hoc analyses were performed following one-way ANOVA to assess pairwise differences for each milestone among the 4 transgender identity groups. The chi-square test was conducted for all other variables to compare frequencies across transgender identity categories, alongside descriptive analyses. For mutually inclusive responses, the chi-square test was conducted separately for each response. All analyses were performed using Stata/MP version 16.0 (StataCorp., College Station, TX, USA).

### Ethics statement

The study was approved by Korea University Institutional Review Board (IRB No. KUIRB-2020-0189-01).

## RESULTS

### Socio-demographic characteristics

Of the 585 participants at Wave 1, 328 (56.1%) were transmasculine and 257 (43.9%) were transfeminine (Table 1). Most participants were in their 20s, with 54.9% aged 19-24 and 25.0% aged 25-29. Regarding education, 61.4% had completed high school or less, while 27.7% had graduated from a 4-year college. In terms of employment, 38.6% were students and 23.9% were unemployed. More than half (55.0%) reported having no monthly income, and 13.2% earned less than 1 million Korean won per month. The majority (71.7%) lived in the greater metropolitan region of Seoul, Gyeonggi-do, and Incheon.

### Developmental milestones for transgender identity

Table 2 presents the mean age for each milestone of transgender identity development, measured at Wave 2. On average, Korean TGNB adults realized their transgender identity at 13.4 years, accepted their transgender identity at 20.1 years, and first disclosed their identity to others (“came out”) at 21.1 years. Based on Bonferroni post hoc tests, binary transgender individuals became aware of their transgender identity earlier than non-binary individuals. Trans men had the youngest average age of acceptance of their transgender identity. Differences in the age of coming out were not statistically significant.

### Experiences in secondary schools

Based on data from Wave 1, among the 579 participants who attended secondary schools, most reported difficulties related to their transgender identity due to the school environment and policies (Table 3). Within the sex-segregated school system, participants were often required to use facilities based on their assigned sex, specifically restrooms (51.5%) and locker rooms (45.8%). Additionally, some were assigned single-sex uniforms (62.2%), schools (37.8%), classes (19.9%), and dormitories (18.0%) that did not align with their gender identity. Transmasculine participants reported greater difficulty with wearing gendered uniforms and the lack of comprehensive sex education in schools, while transfeminine participants reported more difficulty regarding assignments to single-sex classes, dormitories, and locker rooms.

More than two-thirds of the 579 participants reported an anti-LGBTQ school climate. Specifically, schools did not provide comprehensive sexual education inclusive of sexual and gender minorities (69.6%), and teachers made homophobic and transphobic remarks during class (67.4%). Moreover, some participants experienced anti-transgender victimization from teachers, including verbal abuse (13.5%), outing (4.7%), and sexual harassment or violence (4.7%).

### Experiences in the military

Table 4 presents the experiences of 107 trans women and non-binary AMAB participants who had completed or were currently serving their mandatory military service at Wave 1. More than half of these participants (trans women: 52.6%, non-binary AMAB individuals: 58.6%) reported difficulties due to the anti-LGBTQ culture in the military. Additionally, 52.3% feared being outed, and 33.6% feared sexual harassment and violence. Experiences of sexual harassment and violence were reported by 12.1%. Regarding disadvantages experienced during service, 29.0% were classified as maladjusted soldiers, and 12.1% were sent to specialized camps for soldiers with adjustment issues. Trans women were more likely to report difficulties sharing sleeping areas and showers with other soldiers, fears of being outed, and classification as maladaptive soldiers compared to non-binary AMAB participants. These findings highlight the discomfort, vigilance, and mistreatment experienced by trans women in the Korean military.

### Experiences during job applications

At Wave 1, 467 participants had applied for jobs within the prior 5 years. Many reported difficulties related to their TGNB identity during their job search and interviews (Table 5). Specifically, 57.0% had experiences discontinuing their job search and application process due to their identity, while 16.1% had done so due to fears regarding gender recognition history revealed in their documents. Additionally, 36.8% reported difficulty arising from discordance between their legal gender and gender expression, and 26.8% faced challenges with submitting documents that could reveal their TGNB identity. Among those who did apply, 48.0% received negative feedback about their gender expression. Furthermore, 15.6% had hiring decisions rescinded once employers discovered their transgender identity. Compared to transmasculine participants, transfeminine participants were more likely to report greater difficulties during the application process and cancellation or refusal of job offers due to their transgender identity.

### Experiences with gender-affirming healthcare

Table 6 presents the barriers faced by TGNB individuals regarding GAMI, along with the top 3 reasons for not receiving these interventions reported at Wave 1. Approximately one-half of the participants had not undergone the psychiatric evaluations and diagnoses required to commence GAMI. Trans women had the highest proportion of participants with psychiatric diagnoses. Of 584 participants, 4.6% had discontinued hormone treatment, and 52.7% had never received it. Trans women had the highest rate of receiving hormone treatment, while non-binary AFAB participants had the highest rate of non-use. Among all participants, 79.3% had not undergone gender-affirming surgery. Non-binary AFAB individuals most frequently reported no history of gender affirming surgery. For all types of GAMI, financial burden was listed among the top 3 reasons for non-use.

## DISCUSSION

### Key findings

**Figure 1.**
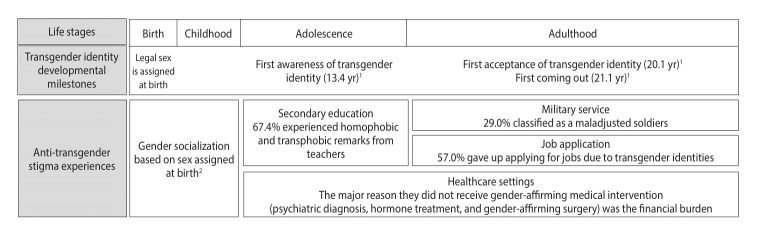
Transgender identity developmental milestones for transgender identity and experiences of anti-transgender stigma from a lifecourse perspective in Korea. ^1^Reported ages represent average ages at each milestone among transgender adults in Korea. ^2^Although children also explore their gender identities and may experience anti-transgender stigma, this study did not investigate these experiences; Future research should address pre-adolescent experiences of anti-transgender stigma.

This study provides the first comprehensive examination of transgender identity developmental milestones in Korea. On average, Korean TGNB individuals realized their transgender identity at age 13, accepted their identity by age 20, and first came out a year later at age 21, timing similar to findings from a United States study [6]. Our findings echo previous research examining the first age of experiencing gender incongruence among TGNB individuals, which reported the lowest average ages among trans men, followed by trans women and non-binary individuals [18]. Additionally, the present study documented the experiences of TGNB individuals in key environments and settings from school years through adulthood, highlighting unique stressors. These experiences of anti-transgender stigma are not isolated but rather are closely intertwined throughout TGNB individuals’ lives, cumulatively impacting their health [3]. This study suggests that stigma in environments experienced during specific stages of identity development may be particularly impactful. The main findings are summarized in Figure 1.

Despite the pervasiveness of anti-transgender stigma across multiple layers and settings of the Korean social structure, laws and policies recognizing and protecting TGNB individuals are sparse. There is yet a formal law on legal gender recognition, signifying a critical institutional oversight. Currently, legal gender recognition for TGNB Koreans is managed according to a Supreme Court regulation, which serves only as a guideline for judges [19]. Additionally, the National Human Rights Commission of Korea Act is the sole legislation explicitly prohibiting discrimination based on gender identity. However, this act has been criticized for its lack of binding authority, as the corrective orders issued by the Commission are not legally enforceable [20]. Policies tailored to settings such as education, the military, the labor market, and healthcare are non-existent or still in early development stages. In the following sections, we outline policy implications based on the findings of the present study to promote equality for TGNB individuals and, consequently, improved health and well-being.

#### Policy implications for the secondary education system

Schools play a crucial role in gender socialization, as children and adolescents interact with teachers and peers who inform their understanding of gender norms and shape their gender expectations [21,22]. Research indicates that TGNB individuals often recognize their gender identity during childhood [23,24] and face victimization and bullying from peers at school [11,25], which contribute to adverse mental health outcomes, including depression and suicidality [11,25]. Our findings align with previous research. The average ages at which transgender identity developmental milestones occur suggest that the period between awareness and acceptance of one’s transgender identity typically coincided with adolescence. Given the critical role of secondary schools for Korean adolescents, policy interventions targeting the secondary education system are fundamental to fostering healthy gender socialization and development among TGNB students in Korea. Our documentation of transgender identity-related victimization experienced by TGNB individuals further underscores the urgent need for inclusive school environments.

A key area requiring improvement and intervention in schools is the practice of sex segregation, underscored by the present findings on hardships faced by TGNB individuals in secondary education settings. Our results indicate that anti-transgender stigma is institutionalized within the Korean secondary school system, especially through single-gender institutions, sex-segregated facilities, and the use of gender-specific uniforms. Studies of TGNB youths in United States secondary schools found that restrictions on restroom and locker room use based on birth-assigned sex were associated with increased prevalence of depression and suicidality [26] and greater likelihood of sexual assault [27]. These findings emphasize the importance of ensuring unrestricted access to sex-segregated facilities for TGNB students, providing gender-neutral options for these facilities, and eliminating discrimination and violence against these students, especially those perpetrated by teachers.

Policies addressing anti-transgender discrimination in schools are also necessary. As of 2023, seven cities and provinces had implemented student human rights ordinances, 5 of which explicitly included sexual orientation and gender identity as protected categories [28]. However, students remained largely unaware of ordinance content, indicating inadequate human rights education in the schools [28]. Coupled with the frequent experiences of anti-transgender stigma reported in the current study, these findings stress the need for increased regional-level and school-level efforts to inspect and monitor adherence to the ordinances. Notably, the reach of these policies and practices remains limited to their specific provincial and institutional contexts. Ideally, these localized efforts should serve to complement national anti-discrimination laws and policies, which are still absent in Korea [10,29].

In conjunction with these policy-level efforts, human rights values can also be incorporated into school curricula, empowering teachers and school leaders to actively create safer environments for TGNB students. Formal curricula may be revised to include age-appropriate content on sexuality and gender diversity, supplemented by informal educational materials [30]. Schools can also provide comprehensive sex education to students, equipping them with knowledge and sensitivity regarding social and health issues related to sex, sexuality, and gender [31] and thus enhancing overall student well-being [32]. Although existing studies were not specific to transgender-focused curriculum materials and TGNB students, their results suggest that interventions aimed at minority student groups can benefit the broader student population.

#### Policy implications for the military

Upon graduation from secondary school, Korean AMAB young adults encounter yet another societal structure built upon patriarchal gender roles and expectations—the military. The Korean military comprises both career soldiers, who are volunteers, and conscripts, who fulfill 2-year mandatory military service required of all able-bodied men between 19 years and 35 years old [33]. While a legal gender change to female can exempt individuals from mandatory service, financial and social barriers often prevent the completion of medical transition and subsequent legal gender recognition by early adulthood [33]. Consequently, many Korean trans women and non-binary AMAB individuals have no choice but to be conscripted after graduating from high school. Given that military service coincides with formative periods in transgender identity development, serving in the military poses unique challenges for Korean trans women and non-binary AMAB individuals.

Our study revealed prevalent reports of experiences related to anti-transgender stigma in the military among trans women and non-binary AMAB individuals. Even in the United States, a country with an all-volunteer force, 93% of transgender soldiers reported bullying and stigma related to their gender identity [34]. The same study indicated that experiences of stigmatization in the military were associated with poor mental health [34]. Additionally, sexual violence has been found to exacerbate mental health issues among sexual and gender minority service members [35]. In Korea, the magnitude of this association may be even greater due to the conscription system, which can force individuals into military service against their will.

The discharge of an openly transgender serviceperson while simultaneously conscripting other TGNB individuals, often against their wishes, highlights an ironic double standard in the Korean military. Moreover, our results showed that TGNB soldiers were often labeled as maladjusted, flagging them for extra attention from officers, with some even being sent to camps for adjustment issues. Although these military policies and practices were intended to be protective, being labeled as maladjusted was inherently stigmatizing. This stigma was intensified by the transgender-specific challenges faced by soldiers in a male-dominated environment during mandatory military service.

The Korean military must acknowledge the transphobia embedded in its policies and culture, recognize it as an occupational hazard for the health of transgender servicepersons, and work towards eradicating anti-transgender stigma and discrimination [36]. Additionally, the exemption criteria in military physical exams used for conscription should be amended to accommodate the realities of TGNB young adults who have not undergone GAMI due to systematic barriers in Korean society.

#### Policy implications for equitable hiring practices

Work is an essential component of life, providing key opportunities to accumulate financial and social resources. However, TGNB individuals in Korea frequently face employment barriers rooted in cisnormative workplace cultures [37]. Research from other countries, primarily the United States, has demonstrated that transgender identity can obstruct individuals from securing interviews or job offers, contributing to high unemployment rates within transgender populations [16,37]. Our findings highlight the employment challenges faced by Korean TGNB individuals, consistent with a systematic review of the international literature that reported that transgender employees frequently experience discrimination, harassment, and stigma [15].

Korean TGNB individuals encounter barriers even before entering the workforce. Many reported discontinuing the process of job search and application out of fear that their gender identity or gender recognition history would be disclosed against their will. Additionally, they experienced difficulties stemming from discordance between their legal gender status and their gender expression, which may be attributed to the lack of transgender visibility and rigid patriarchal gender norms within Korean society. Furthermore, some were entirely barred from workforce entry through denial of job offers based on their transgender identity.

Although the Equal Employment Opportunity and Work-Family Balance Assistance Act and the Labor Standards Act prohibit workplace and hiring discrimination based on sex, these laws provide limited protection for TGNB individuals. Strict measures restricting employers’ access to personal information, including education history and legal gender change, are necessary. To address gaps in existing anti-discrimination policies, the enactment of a comprehensive anti-discrimination law explicitly inclusive of gender identity has been suggested [10,29]. Such legislation should clearly define and prohibit discrimination throughout employment, from hiring to termination. Additionally, enacting a legal gender recognition law that allows TGNB individuals to align their legal gender markers with their current gender identity and presentation, along with provisions protecting their gender recognition history during the hiring process, could provide greater safety for TGNB job seekers [19].

#### Policy implications for inclusive healthcare services

GAMI, which helps align TGNB individuals’ physical characteristics with their gender identity [38], is a prerequisite for legal gender recognition in Korea. Thus, it is crucial for TGNB individuals who desire to undergo legal transition. While international trends are increasingly minimizing psychiatric requirements and depathologizing transgender identities [38,39], Korean TGNB individuals are still required to undergo psychiatric evaluation before accessing GAMI, resulting in additional costs and delays in treatment. Moreover, despite Korea’s advanced medical infrastructure, accessing GAMI is complicated and expensive because it is not covered by the nation’s universal healthcare system [40,41]. Out-of-pocket expenses for these surgical procedures range from 1 million to 60 million Korean Won (approximately 714 to 42,823 US dollar, as of 2025) [42]. These estimates do not account for living expenses during recovery or the overseas travel costs incurred by individuals who opt for surgery abroad due to a lack of qualified healthcare professionals capable of performing gender-affirming surgery in Korea [42].

Our findings confirm that the cost of GAMI represents the primary barrier preventing TGNB individuals from accessing these essential medical interventions, which may be key to leading their authentic lives as transgender individuals. Results from this study indicate that over 70% of participants did not undergo gender-affirming surgery due to financial constraints. This aligns with findings from other countries, including the United States, Canada, Australia, and the United Kingdom [43,44]. Systematic reviews consistently identify financial costs as one of the main barriers to accessing gender-affirming healthcare services [43,44]. Furthermore, a United States study found that income disparities among TGNB patients impact not only whether they undergo gender-affirming surgery but also how early in life they access these services, often relying on private insurance [45]. Korean TGNB individuals likely face similar challenges. Although transgender identity often develops during adolescence and early adulthood, individuals are unlikely to independently amass sufficient financial resources to afford GAMI by their early 20s or even later, especially given the structural exclusion of TGNB individuals from the job market, as discussed previously.

Findings from our study and prior research highlight the urgent importance of addressing the unmet healthcare needs of TGNB individuals resulting from socioeconomic disparities. One potential intervention discussed in the literature is improving coverage of GAMI through private insurance, particularly as many studies originate from the United States [43]. However, in Korea—a country with a universal healthcare system—relying on private insurance coverage could exacerbate income disparities in accessing GAMI. Therefore, coverage should instead be expanded through the National Health Insurance Service to include gender-affirming healthcare services [40]. Additionally, establishing grants and social loan programs at the regional and community levels is recommended to further support TGNB individuals financially, promoting equitable access to necessary healthcare services.

TGNB Koreans face significant challenges not only due to a lack of affordable GAMI but also because of legal misrepresentation and limited financial and social resources [40,46]. Currently, only 1 medical school in Korea offers regular courses specifically addressing LGBTQ health. This gap underscores the need for more inclusive medical education to improve the knowledge, attitudes, and comfort levels of healthcare professionals in treating LGBTQ patients [47]. To address these issues, we recommend increased investments in training healthcare professionals to become competent GAMI providers and creating inclusive healthcare environments for TGNB patients. This includes providing gender-neutral identification options, inclusive administrative paperwork, and accessible physical spaces [48].

#### Overall implications for Korean society

Despite the pervasive anti-transgender stigma documented across multiple social settings in this study, protective measures against transphobic discrimination and victimization in Korea are notably absent at both national and institutional levels. The consistent exposure to anti-transgender stigma across various sectors suggests that such stigma is deeply embedded within Korean society. The systemic nature of this discrimination indicates that policy implications identified for specific settings may have broader applicability throughout Korean society. We recommend developing comprehensive guidelines for gender diversity, implementing cultural competency training tailored to specific environments, and establishing anti-discrimination laws and policies that explicitly protect gender identity and expression at both national and setting-specific levels [29].

Furthermore, legislation of gender recognition laws [19] and comprehensive anti-discrimination law [10,20] are urgently required to ensure that TGNB individuals can align their legal gender status with their gender identity and experience protection from transphobic discrimination. Upon implementation of the suggested trans-inclusive policies and practices, future studies should investigate their impact on the lived experiences of TGNB individuals in Korea and provide comparative analyses with other countries to examine transgender inclusion within Korean society.

### Limitations and strengths

This study has several notable strengths, including its provision of the first nationwide examination of TGNB milestones and experiences of stigma across life stages in Korea, as well as a comprehensive analysis of barriers in multiple institutional settings. However, several limitations must be acknowledged. This study used non-probability convenience sampling, in which participants were recruited from online communities for TGNB individuals, clinics providing GAMI, LGBTQ human rights organizations, and offline LGBTQ-related venues and events. Consequently, TGNB individuals more connected to the TGNB and broader LGBTQ communities were more likely to participate, introducing potential selection bias and limiting the generalizability of the findings. For example, compared to non-participants, study participants might have had greater awareness of anti-transgender discrimination due to their stronger community connections, enabling them to more acutely sense discrimination and impacting their self-report responses. Additionally, the online administration of the survey potentially skewed the sample towards younger adults, limiting insight into the lived experiences of TGNB individuals aged 40 and older. To address potential selection bias, future studies should design their recruitment strategies to include TGNB individuals who are less connected to LGBTQ communities, as well as older TGNB adults, thus increasing generalizability. One approach to overcoming these limitations is to collect and utilize data regarding TGNB individuals from a nationally representative probability sample. This can be achieved through the inclusion of items that measure transgender identity into nationally representative surveys in Korea, which currently lack such items [49]. This would significantly increase generalizability and improve the current understanding of the scope and impact of anti-transgender stigma in Korean society.

For the first time in Korea, this study explored key developmental milestones for transgender identity among TGNB individuals and examined the stigma and discrimination they face in critical settings from adolescence onward. The findings document experiences of anti-transgender stigma across multiple environments, from education to military service to employment. These results underscore the urgent need for comprehensive anti-discrimination legislation and institutional reforms to safeguard the rights of TGNB individuals and enable them to live authentically in Korea.

## Figures and Tables

**Figure f2-epih-47-e2025032:**
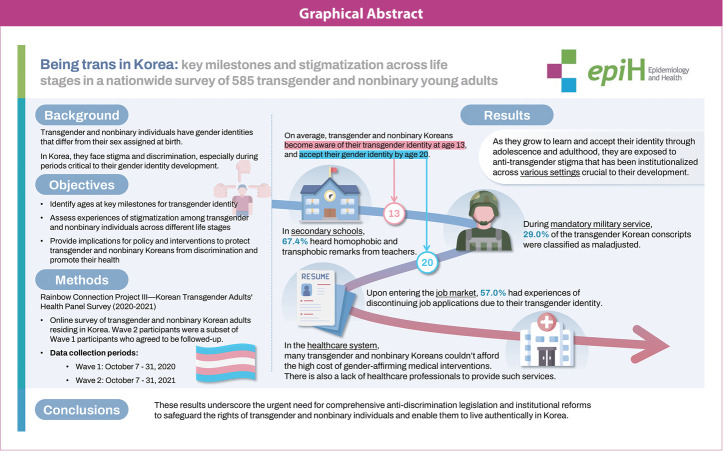


**Table 1.** Socio-demographic characteristics of Korean transgender and non-binary individuals (n=585)

Characteristics	Total	Transgender identity^1^		
		Transmasculine	Transfeminine	p-value^2^
Sexual orientation				<0.001
Heterosexual	106 (18.1)	46 (14.0)	60 (23.3)	
Lesbian or gay	66 (11.3)	23 (7.0)	43 (16.7)	
Bisexual, pansexual	238 (40.7)	131 (39.9)	107 (41.6)	
Asexual	138 (23.6)	104 (31.7)	34 (13.2)	
Other	37 (6.3)	24 (7.3)	13 (5.1)	
Age (yr)				<0.001
19-24	321 (54.9)	201 (61.3)	120 (46.7)	
25-29	146 (25.0)	79 (24.1)	67 (26.1)	
30-34	68 (11.6)	32 (9.8)	36 (14.0)	
35-39	36 (6.2)	14 (4.3)	22 (8.6)	
≥40	14 (2.4)	2 (0.6)	12 (4.7)	
Highest education level attained				0.084
High school or less	359 (61.4)	215 (65.5)	144 (56.0)	
2-Year college	42 (7.2)	18 (5.5)	24 (9.3)	
4-Year college	162 (27.7)	83 (25.3)	79 (30.7)	
Graduate school	22 (3.8)	12 (3.7)	10 (3.9)	
Employment status				0.003
Student	226 (38.6)	148 (45.1)	78 (30.4)	
Employed	157 (26.8)	75 (22.9)	82 (31.9)	
Self-employed or employer	62 (10.6)	34 (10.4)	28 (10.9)	
Unemployed	140 (23.9)	71 (21.6)	69 (26.8)	
Monthly income (10^4^ Korean won)				0.004
None	322 (55.0)	195 (59.5)	127 (49.4)	
<100	77 (13.2)	48 (14.6)	29 (11.3)	
100-199	96 (16.4)	49 (14.9)	47 (18.3)	
200-299	57 (9.7)	25 (7.6)	32 (12.5)	
≥300	33 (5.6)	11 (3.4)	22 (8.6)	
Region				0.485
Seoul	225 (38.5)	120 (36.6)	105 (40.9)	
Gyeonggi-do	159 (27.2)	98 (29.9)	61 (23.7)	
Incheon	35 (6.0)	18 (5.5)	17 (6.6)	
Other metropolitan cities	81 (13.8)	47 (14.3)	34 (13.2)	
Other non-metropolitan areas	85 (14.5)	45 (13.7)	40 (15.6)	
Data collection channel				<0.001
Online advertisement	151 (25.8)	92 (28.0)	59 (23.0)	
Health care clinic	69 (11.8)	18 (5.5)	51 (19.8)	
LGBTQ rights organization	97 (16.6)	63 (19.2)	34 (13.2)	
Friend or acquaintance	202 (34.5)	109 (33.2)	93 (36.2)	
Other	66 (11.3)	46 (14.0)	20 (7.8)	
Total	585 (100)	328 (56.1)	257 (43.9)	

Values are presented as number (%). LGBTQ, lesbian, gay, bisexual, transgender, and queer.

1 Transmasculine refers to individuals assigned female at birth with masculine identities, while transfeminine refers to individuals assigned male at birth with feminine identities.

2 From chi-square tests examining associations of transgender identity by sex assigned at birth with socio-demographic characteristics and data collection channel.

**Table 2.** Milestones in transgender identity development among Korean transgender and non-binary individuals (n=318)^1^

Transgender identity developmental milestones	Total (n=318)	Transgender identity				
		Trans men (n=51)	Trans women (n=102)	Non-binary AFAB (n=126)	Non-binary AMAB (n=39)	p-value^2^
Age of transgender identity (yr)	13.4±5.6	10.5±4.3	12.6±5.6	14.5±5.1	15.6±6.8	<0.001
Age of acceptance of transgender identity (yr)	20.1±5.7	17.4±4.5	20.8±6.4	20.2±4.9	21.1±6.8	0.002
Age of first coming out as transgender (yr)	21.1±6.1	19.9±3.7	22.4±7.1	20.4±5.3	21.7±7.3	0.034

Values are presented as mean±standard deviation. AFAB, assigned female at birth; AMAB, assigned male at birth.

1 Transgender identity developmental milestones were measured in the second wave of the Rainbow Connection Project III—Korean Transgender Adults’ Health Panel Survey, which included 321 participants; The table excludes 3 participants who did not respond to this item.

2 From analyses of variance comparing ages at each transgender identity developmental milestones by transgender identity.

**Table 3.** Experiences of anti-transgender stigma during secondary school years among Korean transgender and non-binary individuals (n=579)^1^

Variables	Total	Transgender identity^2^		
		Transmasculine (n=325)	Transfeminine (n=254)	p-value^3^
Hardships related to gendered school environment				
Attendance of single-sex schools	219 (37.8)	122 (37.5)	97 (38.2)	0.873
Assignment to single-sex classes	115 (19.9)	54 (16.6)	61 (24.0)	0.027
Assignment to single-sex dormitories	104 (18.0)	45 (13.8)	59 (23.2)	0.005
Having to wear gendered uniforms	360 (62.2)	228 (70.2)	132 (52.0)	<0.001
Using restrooms based on birth-assigned sex	298 (51.5)	165 (50.8)	133 (52.4)	0.704
Lack of locker rooms to change clothes	265 (45.8)	124 (38.2)	141 (55.5)	<0.001
Anti-LGBTQ school climate				
No comprehensive sexual education	403 (69.6)	245 (75.4)	158 (62.2)	0.001
Homophobic and transphobic remarks by teachers during class	390 (67.4)	229 (70.5)	161 (63.4)	0.072
Anti-transgender victimization by teacher				
Verbal abuse	78 (13.5)	45 (13.8)	33 (13.0)	0.765
Outing	27 (4.7)	15 (4.6)	12 (4.7)	0.951
Sexual harassment/violence	27 (4.7)	11 (3.4)	16 (6.3)	0.099
Unfair treatment	11 (1.9)	4 (1.2)	7 (2.8)	0.182
Physical violence	8 (1.4)	2 (0.6)	6 (2.4)	0.074

Values are presented as number (%). LGBTQ, lesbian, gay, bisexual, transgender, and queer.

1 Includes only participants who reported attending secondary school.

2 Transmasculine refers to individuals assigned female at birth with masculine identities, while transfeminine refers to individuals assigned male at birth with feminine identities.

3 From chi-square tests examining associations between participants’ transgender identity by sex assigned at birth and their secondary school experiences.

**Table 4.** Experiences of anti-transgender stigma during mandatory military service among Korean trans women and non-binary individuals AMAB (n=107)^1^

Variables	Total	Transgender identity		
		Trans women (n=78)	Non-binary AMAB (n=29)	p-value^2^
Hardships related to male-only military environment				
Homophobic and transphobic culture	58 (54.2)	41 (52.6)	17 (58.6)	0.576
Communal shower room	62 (57.9)	53 (67.9)	9 (31.0)	0.001
Communal sleeping area	47 (43.9)	39 (50.0)	8 (27.6)	0.038
Experiences of violence				
Fear of being outed	56 (52.3)	46 (59.0)	10 (34.5)	0.024
Fears of sexual harassment and violence	36 (33.6)	27 (34.6)	9 (31.0)	0.727
Sexual harassment/violence	13 (12.1)	8 (10.3)	5 (17.2)	0.326
Discrimination during service				
Discriminatory duty assignment	10 (9.3)	9 (11.5)	1 (3.4)	0.201
Classified as a maladjusted soldier	31 (29.0)	27 (34.6)	4 (13.8)	0.035
Sent to specialized camp for soldiers with adjustment issues	13 (12.1)	11 (14.1)	2 (6.9)	0.311
Forced medical examination or hospitalization	5 (4.7)	4 (5.1)	1 (3.4)	0.714
Coercion of forceful discharge	5 (4.7)	5 (6.4)	0 (0)	0.163

Values are presented as number (%). AMAB, assigned male at birth.

1 Of the 257 participants assigned male at birth, 107 reported currently serving or having completed mandatory military service.

2 From chi-square tests examining associations between participants’ transgender identity and their experiences in the military.

**Table 5.** Experiences of anti-transgender stigma during the job application process among Korean transgender and non-binary individuals (n=467)^1^

Variables	Total	Transgender identity^2^		
		Transmasculine (n=261)	Transfeminine (n=206)	p-value^3^
Gave up applying for jobs due to…				
Transgender or non-binary gender identity	266 (57.0)	141 (54.0)	125 (60.7)	0.149
Fear of history of gender transition revealed on documents	75 (16.1)	37 (14.2)	38 (18.4)	0.212
Experienced difficulty due to…				
Discordance between legal gender and gender expression	172 (36.8)	89 (34.1)	83 (40.3)	0.168
Submitting documents that may reveal gender identity	125 (26.8)	53 (20.3)	72 (35.0)	<0.001
Experienced hiring discrimination due to…				
Received negative feedback for not being manly/womanly	224 (48.0)	131 (50.2)	93 (45.1)	0.278
Cancellation or refusal of employment	73 (15.6)	26 (10.0)	47 (22.8)	<0.001

Values are presented as number (%).

1 Includes only participants who reported applying for jobs in the 5 years prior to the first survey.

2 Transmasculine refers to individuals assigned female at birth with masculine identities, while transfeminine refers to individuals assigned male at birth with feminine identities.

3 From chi-square tests examining associations between participants’ transgender identity by sex assigned at birth and their job application experiences.

**Table 6.** Use and reasons for non-use of gender-affirming healthcare among Korean transgender and non-binary individuals

Variables	Total	Transgender identity				
		Trans men	Trans women	Non-binary AFAB	Non-binary AMAB	p-value^1^
Psychiatric diagnosis required to receive gender-affirming medical interventions (n=585)						<0.001
Yes	289 (49.4)	69 (63.9)	156 (83.4)	24 (10.9)	40 (57.1)	
No	296 (50.6)	39 (36.1)	31 (16.6)	196 (89.1)	30 (42.9)	
Reasons for not receiving a psychiatric diagnosis^2^						
No current need for the diagnosis	133 (44.9)	18 (46.2)	7 (22.6)	101 (51.5)	7 (23.3)	0.002
Financial burden	101 (34.1)	16 (41.0)	8 (25.8)	69 (35.2)	8 (26.7)	0.453
No desire for the diagnosis	99 (33.4)	7 (17.9)	5 (16.1)	75 (38.3)	12 (40.0)	0.012
Hormone treatment (n=584)^3^						<0.001
Currently receiving	249 (42.6)	59 (55.1)	143 (76.5)	16 (7.3)	31 (44.3)	
Discontinued	27 (4.6)	6 (5.6)	15 (8.0)	1 (0.5)	5 (7.1)	
Never received	308 (52.7)	42 (39.3)	29 (15.5)	203 (92.3)	34 (48.6)	
Reasons for not receiving hormone treatment^4^						
Financial burden	171 (51.0)	23 (47.9)	22 (50.0)	109 (53.4)	17 (43.6)	0.673
Fear of people’s gaze	140 (41.8)	25 (52.1)	14 (31.8)	84 (41.2)	17 (43.6)	0.264
Had not received the psychiatric diagnosis necessary for hormone prescription	118 (35.2)	21 (43.8)	14 (31.8)	69 (33.8)	14 (35.9)	0.586
Gender-affirming surgery (n=585)						<0.001
Yes	121 (20.7)	45 (41.7)	53 (28.3)	12 (5.5)	11 (15.7)	
No	464 (79.3)	63 (58.3)	134 (71.7)	208 (94.5)	59 (84.3)	
Reasons for not receiving gender-affirming surgery^5^						
Financial burden	328 (70.7)	45 (71.4)	111 (82.8)	134 (64.4)	38 (64.4)	0.002
Just not ready now	175 (37.7)	24 (38.1)	42 (31.3)	80 (38.5)	29 (49.2)	0.130
Expectation of increased difficulty with economic activity	154 (33.2)	27 (42.9)	43 (32.1)	63 (30.3)	21 (35.6)	0.299

Values are presented as number (%). AFAB, assigned female at birth; AMAB, assigned male at birth.

1 From chi-square tests examining associations between participants’ transgender identity by sex assigned at birth and their utilization of gender-affirming medical interventions, as well as reasons for non-utilization.

2 Percentages calculated based on individuals who did not receive a psychiatric diagnosis required for gender-affirming medical interventions.

3 One participant did not complete the portion of the Wave 1 survey regarding hormone usage.

4 Percentages calculated based on individuals not undergoing hormone treatment.

5 Percentages calculated based on individuals who had not received gender-affirming surgery.

## Supplementary materials

Supplementary Material 1. Flow chart of study population.
