# Supplementary figures and images for: Being trans in Korea: key milestones and stigmatization across life stages in a nationwide survey of 585 transgender and non-binary young adults
Source: Epidemiol Health. 2025 Jun 27;47:e2025032. doi: 10.4178/epih.e2025032 (PMC12425868; doi:10.4178/epih.e2025032)

Supplementary material 1. Flow chart of study population


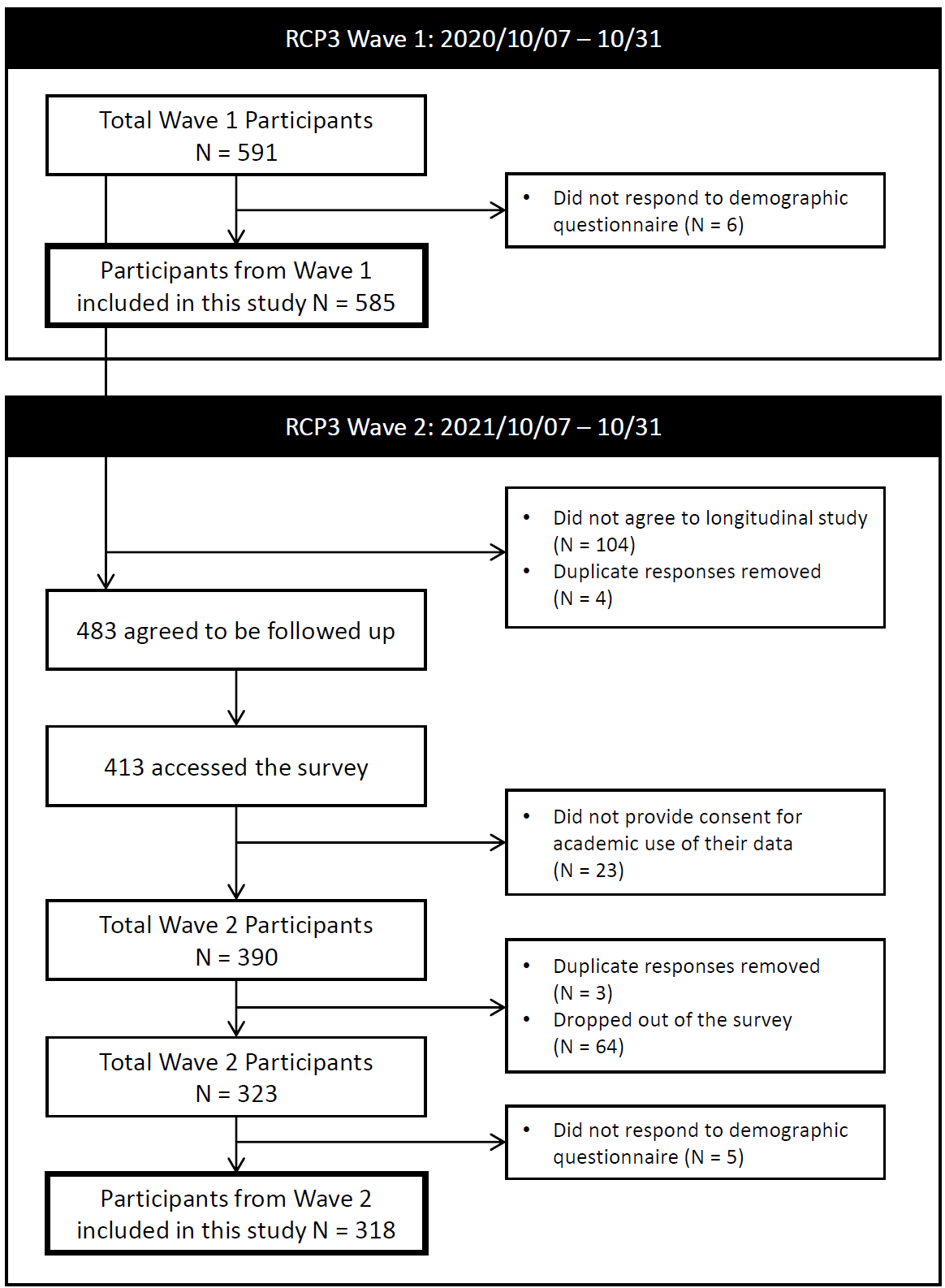

Supplement: Supplementary Material 1. — Flow chart of study population. [file epih-47-e2025032-Supplementary-1.docx]
